# Supplementary material for: Cooperation of N- and C-terminal substrate transmembrane domain segments in intramembrane proteolysis by γ-secretase
Source: Commun Biol. 2023 Feb 15;6:177. doi: 10.1038/s42003-023-04470-5 (PMC9931712; doi:10.1038/s42003-023-04470-5)
Supplement: Supplementary file 2 — Supplementary Material [file 42003_2023_4470_MOESM2_ESM.pdf]

Supplementary Information

Cooperation of N- and C-terminal substrate transmembrane domain segments in intramembrane proteolysis by γ-secretase

Nadine T. Werner[1§], Philipp Högel[2§], Gökhan Güner[3§], Walter Stelzer[2], Manfred Wozny[4], Marlene Aßfalg[3,5], Stefan F. Lichtenthaler[3,5,6*], Harald Steiner[1,5,6*], and Dieter Langosch[2*]

[1] Biomedical Center (BMC), Division of Metabolic Biochemistry, Faculty of Medicine, LMU Munich, Munich, Germany

[2] Chair of Biopolymer Chemistry, Technical University of Munich, Freising, Germany

[3] German Center for Neurodegenerative Diseases (DZNE), Munich, Germany

[4] Am Goldhammer 11, 90491 Nürnberg, Germany

[5] Neuroproteomics, School of Medicine, Klinikum rechts der Isar, Technical University of Munich, Munich, Germany

[6] Munich Cluster for Systems Neurology (SyNergy), Munich, Germany

[§] These authors contributed equally

[*] Corresponding authors:

D. Langosch, langosch@tum.de

H. Steiner, harald.steiner@med.uni-muenchen.de

S. Lichtenthaler, stefan.lichtenthaler@dzne.de

Supplementary Note 1

The origin of biphasic DHX. To explain the origin of biexponential, or biphasic, DHX, we reiterate that exchange at a given amide within a population of TMDs is dominated by the EX2 regime where the local folding rate vastly exceeds the chemical rate constant, resulting in non-correlated exchange events [1] as previously shown by demonstrating a gradual mass shift of the isotopic envelope of the C99 TMD peptide [2]. In addition to those non-correlated exchange events, we propose that very unstable regions of our TMD helices, such as a glycine-rich hinge or frayed helix termini, may undergo rare correlated exchange of two (or even more) neighboring deuterons, i.e. limited EX1 exchange [3] in addition to the prevalent EX2 regime. Mixed EX1/EX2 modes of amide exchange have been demonstrated previously to describe local protein fluctuations [4,5]. The abundance our peptides having undergone correlated DHX is apparently too low for visibly affecting the isotope pattern produced by the prevalent non-correlated DHX. Nonetheless, the superposition of isotope patterns resulting from EX1 and EX2 exchange occuring in parallel is predicted to mimic slightly reduced average masses at two neighboring positions when applying the fitting procedure (assuming the most likely case of two neighboring correlated exchange events).

This is expected to translate into artificially accelerated DHX kinetics over time. However, accelerated exchange is expected only within the initial periods of the exchange reaction where neighboring amide deuterons are abundant enough for correlated DHX to give rise to correlated exchange of deuterons. At later time points, where ongoing DHX has reduced the numbers of neighboring deuterons, correlated DHX/HDX is expected to replace correlated DHX, thus producing less artificial acceleration of the kinetics. As a result, at later time points of the reaction, we expect kinetics that cannot be distinguished from EX2, allowing us to calculate ΔG values from the data. We note that these slow parts of the biphasic kinetics cover the major fraction in most cases where amide deuterons exhibit biphasic exchange, as reflected by the respective population sizes A (fast part) and B (slow part) (Table S2; see Methods). To illustrate the point, Fig. S2 shows mixed EX1 and EX2 kinetics and its consequences using an idealized model peptide.

In sum, this model is well suited to explain fast initial exchange kinetics followed by slower kinetics at highly flexible regions of a helix. We infer that the slow parts of the kinetics allow us to calculate accurate thermodynamic stabilities at unstable sites. Moreover, we propose that the apparent existence of correlated exchange events may indicate slow backfolding events after local helix unfolding.

Potential impact of experimental conditions on calculated H-bond strength. Exchange rate constants k_{exp} are transformed into ΔG values by relating them to the rates k_{ch} of chemical exchange of unfolded peptides. In general, amides where k_{ch} values from the literature exceed the k_{exp} [6] are protected from exchange depending on their H-bond strength. We note that k_{ch} may be lower under

our conditions than the standard values for several potential reasons: (i) The molarity of water in 80% (v/v) TFE solvent is only 20% of the bulk molarity used for the determination of the reference chemical exchange rates k_{ch}; (ii) the hydration of residues in the hydrophobic core of a TMD dissolved in 80% TFE may be reduced relative to bulk water; (iii) k_{ch} values determined for model tripeptides in the unfolded state may not correctly represent sterical hindrance of exchange in the helical state; and (iv) TFE might have an impact on the autoionization constant of water and k_{ch} [7]. As a result, our calculated ΔG values might be underestimating the true values to some extent.

Supplementary Note 2: Statistics in the Analysis of Amide Exchange Kinetics. It is assumed that exchange data are available for N exchange periods t_n (n=1, 2, …, N). To analyze the exchange kinetics of the amide hydrogen of amino acid m, the first step consists in the determination of the mean numbers D_{mean} (m,n) of the D numbers D_m obtained for all the fragment types analyzed for time point n.

The main model employed for the analysis of the kinetics predicts a first order reaction that leads from the starting value D_{start} of the D number to the asymptotic D number $D_{asymptotic}$. The experimental rate constant $k_{exp,m}$ for the exchange of amide hydrogen m is determined by a non-linear least squares fitting routine that minimizes the quantity χ^2 of equation (1a):

$$\begin{aligned}&\chi^2 = \sum_{n=1}^{N}\left(\chi_{m,n}\right)^2 \text{ with } \chi_{m,n} := D_{mean}(m,n) - D_{fit}(m,n) \text{ and}\\&D_{fit}(m,n) := D_{asymptotic} + \left(D_{start} - D_{asymptotic}\right)\cdot \exp\left(-k_{exp,m}\cdot t_n\right).\end{aligned} \quad (1a)$$

For amide hydrogens exhibiting a biphasic exchange dynamics, the quantity χ^2 of equation (1b) is minimized to determine the rate constants $k_{1\ exp,m}$, $k_{2\ exp,m}$ and the ratio A of the conformation exhibiting the rate constant $k_{1\ exp,m}$:

$$\begin{aligned}&\chi^2 = \sum_{n=1}^{N}\left(\chi_{m,n}\right)^2 \text{ with } \chi_{m,n} := D_{mean}(m,n) - D_{fit}(m,n) \text{ and}\\&D_{fit}(m,n) := D_{asymptotic} + \left(D_{start} - D_{asymptotic}\right)\cdot E,\\&E = A\cdot \exp\left(-k_{1\ exp,m}\cdot t_n\right) + (1-A)\cdot \exp\left(-k_{2\ exp,m}\cdot t_n\right).\end{aligned} \quad (1b)$$

For H/D and D/H exchange, the number D_{start} amounts to 0 and 1, respectively. In case some of the numbers D_{mean}(m,n) are not available due to missing fragments for certain time points, the fitting procedure is restricted to the time points with available D numbers. For the fitting to be performed, the number of time points with D numbers must not be smaller than three and six for monophasic and biphasic behavior, respectively.

To estimate the standard errors of the fitted quantities (monophasic behavior: $k_{exp,m}$, biphasic behavior: $k_{1\ exp,m}$, $k_{2\ exp,m}$ and A), the standard deviation σ_m is calculated for the residuals $\chi_{m,n}$ obtained for the best fit. The standard deviation σ_m is then used to perform the fitting defined in (1a) or (1b) for additional 2N sets of D numbers:

$$\begin{array}{llllll}
\text{Set 1-1:} & D_{mean}(m,1)+\sigma_m & D_{mean}(m,2) & \ldots & D_{mean}(m,N-1) & D_{mean}(m,N)\\
\text{Set 1-2:} & D_{mean}(m,1)-\sigma_m & D_{mean}(m,2) & \ldots & D_{mean}(m,N-1) & D_{mean}(m,N)\\
\ldots & \ldots & \ldots & \ldots & \ldots & \ldots\\
\text{Set n-1:} & D_{mean}(m,1) & \ldots & D_{mean}(m,n)+\sigma_m & \ldots & D_{mean}(m,N)\\
\text{Set n-2:} & D_{mean}(m,1) & \ldots & D_{mean}(m,n)-\sigma_m & \ldots & D_{mean}(m,N)\\
\ldots & \ldots & \ldots & \ldots & \ldots & \ldots\\
\text{Set N-1:} & D_{mean}(m,1) & D_{mean}(m,2) & \ldots & D_{mean}(m,N-1) & D_{mean}(m,N)+\sigma_m\\
\text{Set N-2:} & D_{mean}(m,1) & D_{mean}(m,2) & \ldots & D_{mean}(m,N-1) & D_{mean}(m,N)-\sigma_m
\end{array}$$

The uncertainty $\Delta_{m,n}$ of the decimal logarithm of the rate constant $k_{exp,m}$ that is caused by the uncertainty σ_m of the numbers $D_{mean}(m,n)$ is estimated as the mean of the two numbers |log[$k_{exp,m}$]-log[k(Set n-1)]| and |log[$k_{exp,m}$]-log[k(Set n-2)]|. The quantities k(Set n-1) and k(Set n-2) stand for the best fit rate constants obtained with the sets of D-numbers Set n-1 and Set n-2, respectively. By error propagation, the standard error $\Delta\log(k_{exp,m})$ of the decimal logarithm of the experimental rate constant $k_{exp,m}$ is calculated in the following way:

$$\Delta \log\left(k_{exp,m}\right) = \sum_{n=1}^{N} \Delta_{m,n}. \tag{2a}$$

In case of a biexponential fit, the uncertainties $\Delta_{1\ m,n}$ and $\Delta_{2\ m,n}$ of the decimal logarithms of the rate constants $k_{1\ exp,m}$ and $k_{2\ exp,m}$, respectively, are estimated in full analogy to the estimation of $\Delta_{m,n}$. The standard errors $\Delta\log(k_{1\ exp,m})$ and $\Delta\log(k_{2\ exp,m})$ are calculated in the following way:

$$\Delta \log\left(k_{1\,exp,m}\right) = \sum_{n=1}^{N} \Delta_{1\,m,n},\ \ \Delta \log\left(k_{2\,exp,m}\right) = \sum_{n=1}^{N} \Delta_{2\,m,n}. \tag{2b}$$

To estimate the uncertainty of the relative abundance of the conformation with the rate constant $k_{1\ exp,m}$, the means $\Delta A_{m,n}$ of the two numbers $|A_{,m}$- A(Set n-1)| and $|A_{,m}$- A(Set n-2)| are calculated with A(Set n-1) and A(Set n-2) standing for the ratios obtained with the sets of D-numbers Set n-1 and Set n-2, respectively. By error propagation, the standard error $\Delta A_{,m}$ of the experimental ratio $A_{,m}$ is calculated as follows:

$$\Delta A_{m} = \sum_{n=1}^{N} \Delta A_{m,n}. \tag{3c}$$

In cases where $k_{ch,m}$ is available, the experimental rate constant $k_{exp,m}$ can be used to calculate the difference ΔG_m of the Gibbs Free Energy associated with the equilibrium between the effectively folded and the effectively unfolded situation for amide hydrogen m. If the rate constant of the unfolding reaction of the peptide, which leads to an exchange competent state of amide hydrogen m, is denoted by $k_{m,+}$, and if the rate constant of the reverse reaction that makes the amide hydrogen m not exchange competent is denoted by $k_{m,-}$, ΔG_m is given by equation (4):

$$\Delta G_{m} = -RT\cdot\ln\left(K_{m}\right) = -RT\cdot\ln\left(\frac{k_{m,+}}{k_{m,-}}\right). \tag{4}$$

According to equation (5), the fraction $k_{m,+}/(k_{m,+}+k_{m,-})$ can be determined from the experimental rate constant $k_{exp,m}$ and the chemical rate constant $k_{ch,m}$:

$$\frac{k_{m,+}}{k_{m,+}+k_{m,-}} = \frac{k_{exp,m}}{k_{ch,m}}. \tag{5}$$

Thus, the fraction of rate constants needed for the calculation of ΔG_m by means of equation (4) can be calculated from the experimental rate constant $k_{exp,m}$ and the chemical rate constant $k_{ch,m}$ according to equation (6):

$$\frac{k_{exp,m}}{k_{ch,m}} = \frac{k_{m,+}}{k_{m,+}+k_{m,-}} = \frac{\frac{k_{m,+}}{k_{m,-}}}{\frac{k_{m,+}}{k_{m,-}}+1} \rightarrow \frac{k_{m,+}}{k_{m,-}} \cdot \left(1-\frac{k_{exp,m}}{k_{ch,m}}\right) = \frac{k_{exp,m}}{k_{ch,m}} \rightarrow$$
$$\rightarrow \frac{k_{m,+}}{k_{m,-}} = \frac{k_{exp,m}}{k_{ch,m}-k_{exp,m}}. \quad (6)$$

The limits of the standard confidence interval of ΔG_m are calculated by means of the standard error $\Delta\log(k_{exp,m})$ of the decimal logarithm of $k_{exp,m}$ using equations (7):

$$\Delta G_{m,min} = -RT \cdot \ln\left(\frac{k_{+,m}}{k_{-,m}}\right)_{max} \text{ with } \left(\frac{k_{+,m}}{k_{-,m}}\right)_{max} = \frac{k_{exp,m} \cdot 10^{\Delta\log(k_{exp,m})}}{k_{ch,m}-k_{exp,m} \cdot 10^{\Delta\log(k_{exp,m})}},$$
$$\Delta G_{m,max} = -RT \cdot \ln\left(\frac{k_{+,m}}{k_{-,m}}\right)_{min} \text{ with } \left(\frac{k_{+,m}}{k_{-,m}}\right)_{min} = \frac{k_{exp,m} \cdot 10^{-\Delta\log(k_{exp,m})}}{k_{ch,m}-k_{exp,m} \cdot 10^{-\Delta\log(k_{exp,m})}}. \quad (7)$$

In case of a biexponential fit, instead of being performed with $k_{exp,m}$, the calculation of ΔG_m, $\Delta G_{m,min}$ and $\Delta G_{m,max}$ is based on the smaller of the two rate constants $k_{1\ exp,m}$ and $k_{2\ exp,m}$.

The plausibility of the biexponential fit is tested by means of the hypotheses H0 and H1: H0 means that the exchange behavior is monoexponential, whereas H1 means that it is biexponential. For both the hypotheses H0 and H1, the likelihood values λ0 and λ1 are calculated, respectively. The calculation is based on the assumption that the $D_{mean(m,n)}$ numbers follow Gaussian distributions centered around $D_{fit(m,n)}$ and with standard deviations amounting to σ_m for all exchange periods. The standard deviation σ_m is estimated by means of the residuals of the fitting procedure. As a consequence, the values λ0 and λ1 are calculated according to the equations (8a) and (8b), respectively:

$$\lambda 0 = \prod_{n=1}^{N}\left[\frac{1}{\sqrt{2\pi}\cdot\sigma_m}\cdot\exp\left(-\frac{\chi_{m,n}^2}{2\cdot\sigma_m^2}\right)\right] \text{ with}$$
$$\chi_{m,n} = D_{mean}(m,n) - D_{fit}(m,n) \text{ and } \sigma_m = \sqrt{\frac{\sum_{n=1}^{N}\chi_{m,n}^2}{(N-1)}},$$
$$D_{fit}(m,n) := D_{asymptotic} + \left(D_{start} - D_{asymptotic}\right)\cdot\exp\left(-k_{exp,m}\cdot t_n\right), \quad (8a)$$

$k_{exp,m}$: values obtained by fitting according to (1a)

$$\lambda 1=\prod_{n=1}^{N}\left[\frac{1}{\sqrt{2\pi}\cdot\sigma_m}\cdot\exp\left(-\frac{{\chi_{m,n}}^2}{2\cdot{\sigma_m}^2}\right)\right] \quad \text{with}$$

$$\chi_{m,n}=D_{mean}(m,n)-D_{fit}(m,n) \text{ and } \sigma_m=\sqrt{\frac{\sum_{n=1}^{N}{\chi_{m,n}}^2}{(N-1)}},$$

$$D_{fit}(m,n):=D_{asymptotic}+\left(D_{start}-D_{asymptotic}\right)\cdot E,$$

$$E=A\cdot\exp\left(-k_{1\,exp,m}\cdot t_n\right)+(1-A)\cdot\exp\left(-k_{2\,exp,m}\cdot t_n\right), \qquad (8b)$$

$k_{1exp,m}$, $k_{2exp,m}$ and A: values obtained by fitting according to (1b)

In order to apply Wilks' theorem, the likelihood values λ0 and λ1 are used for the calculation of the test statistic T:

$$T = -2\,ln\left(\frac{\lambda 0}{\lambda 1}\right) \qquad (9)$$

Since the biexponential fit implies three parameters (degrees of freedom d1=3), whereas the monoexponential fit implies only a single parameter (degrees of freedom d0=1), the non-negative likelihood ratio λ0/λ1 is smaller than 1. This means that T is always greater than or equal to zero. In order to decide whether T is not significantly greater than zero, i.e. the hypothesis H0 is to be accepted, the test statistic T is compared with its probability distribution. According to Wilks' theorem, this probability distribution is approximately a χ_2 distribution with degrees of freedom equal to d3-d1=2 ($\chi^2_{d1-d0=2}$). The p-value for the validity of H0 is equal to the probability to observe values of the test statistic greater than or equal to T.

Since $\chi^2_{d1-d0=2}$ is an exponential distribution with the rate parameter 1/2, the p-value (p) for the validity of H0, i.e. a monoexponential behavior is calculated as follows:

$$p=\int_T^{\infty}\chi^2_{d1-d0=2}(x)dx=\int_T^{\infty}2\cdot\exp\left(-\frac{x}{2}\right)dx=\lim_{x\to\infty}\left(-\exp\left(-\frac{x}{2}\right)+\exp\left(-\frac{T}{2}\right)\right)=\exp\left(-\frac{T}{2}\right). \qquad (10)$$

Supplementary Table 1. Sequences of synthetic peptides investigated in this study

Peptide	Sequence
**wt C99**	Ac-KK**KGAIIGLMVGGVVIATVIVITLVMLKKK**-NH_2[1]
**C99 I47G/T48G**	Ac-KK**KGAIIGLMVGGVVIATVIV**GG**LVMLKKK**-NH_2
**C99 I47L/T48L**	Ac-KK**KGAIIGLMVGGVVIATVIV**LL**LVMLKKK**-NH_2
**pL-A9**	Ac-KKKLLLLLLLLLLLLLLLAAAAAAAAAKKK-NH_2
**pL-GG**	Ac-KKKLLLLLLLL**GG**LLLLLLLLLLLLLLKKK-NH_2
**pL-VGGV**	Ac-KKKLLLLLLL**VGG**VLLLLLLLLLLLLLKKK-NH_2
**pL-VGGV/εGG**	Ac-KKKLLLLLLL**VGGV**LLLLLLLLGGLLLKKK-NH_2
**pL-VGGV/cr**	Ac-KKKLLLLLLL**VGGV**LLLL**VIVITLVMLKKK**-NH_2
**ErbB4**	Ac-KKK**LIAAGVIGGLFILVIVGLTFAVYV**KKK-NH_2
**N-Cadherin**	Ac-KKK**GAIIAILLCIIILLILVLMFVVWM**KKK-NH_2
**Notch1**	Ac-KKK**LHFMYVAAAAFVLLFFVGCGVLLS**KKK-NH_2

[1] Termini were blocked by acetylation (N-terminus) or amidation (C-terminus) in order to remove non-natural charges. The natural parts of the sequences shown are in bold face type. Two N-terminal Lys residues were appended to C99 derivatives to enhance solubility and gas phase fragmentation by ETD [8]. Lys tags similar to those of C99 derivatives were appended to ErbB4, N-Cadherin, and Notch1 sequences in order to ensure similar conditions, such as hydration, to all TMDs compared here. We note that similarly constructed C99 TMD peptides were previously shown to be good substrates for γ-secretase [9].

Supplementary Table 2. Exchange rate constants and population sizes

	wt C99 [1]						Notch1 wt [1]				
**Residue number**	**amide**	**log $k_{(B)}$** [2] [min^{-1}]	**log k_A** [3] [min^{-1}]	**fraction A** [4]	**D(0)** [5]	**Residue numnber**	**amide**	**log $k_{(B)}$** [2] [min^{-1}]	**log k_A** [3] [min^{-1}]	**fraction A** [4]	**D(0)** [5]
**3**	K28	n.d.[6]				**3**		n.d.[6]			
**4**	G29	n.d.				**4**	L1734	n.d.			
**5**	A30	n.d.				**5**	H1735	n.d.			
**6**	I31	n.d.				**6**	F1736	n.d.			
**7**	I32	-0.578 ± 0.067	0.630 ± 0.145	0.42 ± 0.06	1.00	**7**	M1737	n.d.			
**8**	G33	-0.160 ± 0.051			1.00	**8**	Y1738	-0.884 ± 0.057	0.506 ± 0.085	0.53 ± 0.03	1.00
**9**	L34	-0.122 ± 0.042			1.00	**9**	V1739	-0.893 ± 0.044			1.00
**10**	M35	-0.272 ± 0.033			1.00	**10**	A1740	-1.045 ± 0.022			1.00
**11**	V36	-0.611 ± 0.049			1.00	**11**	A1741	-1.599 ± 0.139	-0.742 ± 0.144	0.55 ± 0.15	1.00
**12**	G37	-1.388 ± 0.163	-0.242 ± 0.206	0.39 ± 0.13	1.00	**12**	A1742	-1.470 ± 0.041			0.90
**13**	G38	-1.771 ± 0.175	-0.007 ± 0.187	0.35 ± 0.07	1.00	**13**	A1743	-2.622 ± 0.184	-1.300 ± 0.117	0.66 ± 0.08	1.00
**14**	V39	-1.780 ± 0.118	0.265 ± 0.287	0.26 ± 0.05	1.00	**14**	F1744	-2.670 ± 0.095	-1.151 ± 0.266	0.32 ± 0.07	1.00
**15**	V40	-2.214 ± 0.118	-0.978 ± 0.256	0.33 ± 0.11	1.00	**15**	V1745	-2.577 ± 0.045			0.85
**16**	I41	-2.260 ± 0.111	-0.945 ± 0.181	0.35 ± 0.11	1.00	**16**	L1746	-2.744 ± 0.054			0.90
**17**	A42	-2.459 ± 0.044	-0.431 ± 0.277	0.31 ± 0.05	1.00	**17**	L1747	-2.692 ± 0.067			1.00
**18**	T43	-2.647 ± 0.097			0.75	**18**	F1748	-2.777 ± 0.063			0.95
**19**	V44	-3.546 ± 0.223			0.95	**19**	F1749	-2.473 ± 0.051			0.95
**20**	I45	-4.160 ± 0.401			1.00	**20**	V1750	-2.647 ± 0.099	-0.563 ± 0.150	0.50 ± 0.05	1.00
**21**	V46	-4.035 ± 0.279			1.00	**21**	G1751	-1.919 ± 0.162	-0.523 ± 0.261	0.41 ± 0.11	1.00
**22**	I47	-3.892 ± 0.127			1.00	**22**	C1752	-1.521 ± 0.212	0.213 ± 0.624	0.33 ± 0.12	1.00
**23**	T48	-3.625 ± 0.165			1.00	**23**	G1753	-0.877 ± 0.110	1.071 ± 0.312	0.30 ± 0.07	1.00
**24**	L49	-3.704 ± 0.074	-0.466 ± 0.163	0.24 ± 0.03	1.00	**24**	V1754	-1.623 ± 0.121	1.031 ± 0.227	0.52 ± 0.06	1.00
**25**	V50	-3.263 ± 0.127	-0.919 ± 0.263	0.23 ± 0.05	1.00	**25**	L1755	-1.021 ± 0.084	1.067 ± 0.390	0.22 ± 0.06	1.00
**26**	M51	-2.499 ± 0.116			1.00	**26**	L1756	-1.009 ± 0.071	0.990 ± 0.140	0.34 ± 0.04	1.00
**27**	L52	-2.602 ± 0.165	-0.929 ± 0.236	0.46 ± 0.10	1.00	**27**	S1757	-1.175 ± 0.217	0.759 ± 0.210	0.48 ± 0.07	1.00
**28**	K53	n.d.				**28**		n.d.			
**29**	K54	n.d.				**29**		n.d.			
**30**	K55	n.d.				**30**		n.d.			

		ErbB4 [1]						N-Cadherin [1]			
**Residue number**	**amide**	**log $k_{(B)}$** [2] [min^{-1}]	**log k_A** [3] [min^{-1}]	**fraction A** [4]	**D(0)** [5]	**Residue numnber**	**amide**	**log $k_{(B)}$** [2] [min^{-1}]	**log k_A** [3] [min^{-1}]	**fraction A** [4]	**D(0)** [5]
**3**		n.d.[6]				**3**		n.d.[6]			
**4**	L652	n.d.				**4**	G723	n.d.			
**5**	I653	n.d.				**5**	A724	n.d.			
**6**	A654	n.d.				**6**	I725	n.d.			
**7**	A655	0.553 ± 0.068			1.00	**7**	I726	-0.894 ± 0.070	0.212 ± 0.132	0.46 ± 0.06	
**8**	G656	0.519 ± 0.136			1.00	**8**	A727	-1.336 ± 0.098	-0.316 ± 0.177	0.33 ± 0.09	1.00
**9**	V657	-1.158 ± 0.142	0.910 ± 0.175	0.44 ± 0.05	1.00	**9**	I728	-2.061 ± 0.058	-0.757 ± 0.144	0.32 ± 0.05	1.00
**10**	I658	-1.188 ± 0.109	0.676 ± 0.231	0.44 ± 0.04	1.00	**10**	L729	-2.057 ± 0.063			1.00
**11**	G659	-0.776 ± 0.070	0.818 ± 0.136	0.39 ± 0.06	1.00	**11**	L730	-2.282 ± 0.057			1.00
**12**	G660	-0.498 ± 0.078			1.00	**12**	C731	-2.333 ± 0.068			0.90
**13**	L661	-0.898 ± 0.068			1.00	**13**	I732	-2.919 ± 0.195	-1.089 ± 0.495	0.38 ± 0.13	1.00
**14**	F662	-2.727 ± 0.195	-0.812 ± 0.104	0.65 ± 0.05	1.00	**14**	I733	-3.773 ± 0.138	-1.711 ± 0.210	0.36 ± 0.06	1.00
**15**	I663	-3.009 ± 0.114	-0.609 ± 0.073	0.55 ± 0.03	1.00	**15**	I734	-3.856 ± 0.316			0.85
**16**	L664	-2.640 ± 0.127			1.00	**16**	L735	-4.077 ± 0.182			0.90
**17**	V665	-3.325 ± 0.133	-2.276 ± 0.217	0.38 ± 0.13	1.00	**17**	L736	-4.275 ± 0.364			1.00
**18**	I666	-3.493 ± 0.093	-1.820 ± 0.226	0.24 ± 0.05	1.00	**18**	I737	n.d.			
**19**	V667	-3.845 ± 0.169	-2.750 ± 0.151	0.40 ± 0.12	1.00	**19**	L738	-4.746 ± 0.169	-2.678 ± 0.667	0.25 ± 0.09	0.95
**20**	G668	-3.566 ± 0.114	-2.272 ± 0.082	0.56 ± 0.06	1.00	**20**	V739	-4.326 ± 0.145			1.00
**21**	L669	-2.417 ± 0.075			1.00	**21**	L740	-4.835 ± 0.131	-3.074 ± 0.279	0.32 ± 0.11	1.00
**22**	T670	-2.194 ± 0.079			1.00	**22**	M741	-4.369 ± 0.246	-2.571 ± 0.287	0.33 ± 0.09	1.00
**23**	F671	-2.071 ± 0.063			0.85	**23**	F742	-3.773 ± 0.659	-2.040 ± 0.275	0.36 ± 0.12	1.00
**24**	A672	-2.362 ± 0.411	-0.360 ± 0.165	0.54 ± 0.11	1.00	**24**	V743	-3.898 ± 0.222	-2.072 ± 0.316	0.33 ± 0.09	1.00
**25**	V673	-2.049 ± 0.058	-0.259 ± 0.181	0.25 ± 0.04	1.00	**25**	V744	-2.942 ± 0.086			1.00
**26**	Y674	-1.836 ± 0.068			1.00	**26**	W745	-4.136 ± 0.533	-2.209 ± 0.130	0.63 ± 0.10	1.00
**27**	V675	-1.855 ± 0.034	-0.066 ± 0.167	0.21 ± 0.03	1.00	**27**	M746	-2.721 ± 0.225	-1.416 ± 1.378	0.52 ± 0.22	1.00
**28**		n.d.				**28**		n.d.			
**29**		n.d.				**29**		n.d.			
**30**		n.d.				**30**		n.d.			

Residue number	amide	C99 I47G/T48G [1] log $k_{(B)}$ [2] [min^{-1}]	log k_A [3] [min^{-1}]	fraction A [4]	D(0) [5]
**3**	K28	n.d.[6]			
**4**	G29	n.d.			
**5**	A30	n.d.			
**6**	I31	n.d.			
**7**	I32	-0.331 ± 0.021	1.050 ± 0.058	0.28 ± 0.02	1.00
**8**	G33	-0.311 ± 0.072	0.777 ± 0.121	0.40 ± 0.07	1.00
**9**	L34	-0.395 ± 0.051	0.845 ± 0.122	0.29 ± 0.05	1.00
**10**	M35	-0.711 ± 0.104	0.202 ± 0.166	0.50 ± 0.11	1.00
**11**	V36	-1.076 ± 0.089	-0.156 ± 0.103	0.40 ± 0.09	1.00
**12**	G37	-0.773 ± 0.074			1.00
**13**	G38	-1.256 ± 0.082	-0.109 ± 0.134	0.36 ± 0.07	1.00
**14**	V39	-1.721 ± 0.034	0.110 ± 0.209	0.23 ± 0.03	1.00
**15**	V40	-2.231 ± 0.139	-0.087 ± 1.097	0.21 ± 0.09	1.00
**16**	I41	-2.459 ± 0.032	-1.213 ± 0.062	0.28 ± 0.02	1.00
**17**	A42	-2.168 ± 0.072			1.00
**18**	T43	-2.239 ± 0.047			0.95
**19**	V44	-2.747 ± 0.171			0.90
**20**	I45	-3.092 ± 0.392			0.90
**21**	V46	-3.025 ± 0.158			0.90
**22**	**G47**	-2.780 ± 0.162	-1.599 ± 0.125	0.51 ± 0.09	0.85
**23**	**G48**	-1.609 ± 0.074	0.303 ± 0.570	0.19 ± 0.07	1.00
**24**	L49	-1.449 ± 0.039			1.00
**25**	V50	-1.413 ± 0.042			1.00
**26**	M51	-1.313 ± 0.066			1.00
**27**	L52	-1.304 ± 0.042	0.926 ± 0.133	0.27 ± 0.02	1.00
**28**	K53	n.d.			
**29**	K54	n.d.			
**30**	K55	n.d.			

Residue numnber	amide	C99 I47L/T48L [1] log $k_{(B)}$ [2] [min^{-1}]	log k_A [3] [min^{-1}]	fraction A [4]	D(0) [5]
**3**	K28	n.d.[6]			
**4**	G29	n.d.			
**5**	A30	n.d.			
**6**	I31	n.d.			
**7**	I32	-0.372 ± 0.128	0.769 ± 0.113	0.54 ± 0.09	1.00
**8**	G33	-0.469 ± 0.071	0.666 ± 0.048	0.59 ± 0.04	1.00
**9**	L34	-0.460 ± 0.071	0.838 ± 0.098	0.45 ± 0.05	1.00
**10**	M35	-0.471 ± 0.060	0.878 ± 0.150	0.32 ± 0.06	1.00
**11**	V36	-0.536 ± 0.084			1.00
**12**	G37	-0.536 ± 0.119			1.00
**13**	G38	-1.131 ± 0.061	0.165 ± 0.099	0.37 ± 0.04	1.00
**14**	V39	-1.709 ± 0.084	-0.433 ± 0.108	0.32 ± 0.06	1.00
**15**	V40	-1.974 ± 0.085	-0.217 ± 0.952	0.12 ± 0.06	1.00
**16**	I41	-1.986 ± 0.177			0.95
**17**	A42	-1.931 ± 0.107			1.00
**18**	T43	-2.857 ± 0.399	-1.298 ± 0.597	0.48 ± 0.21	1.00
**19**	V44	-3.191 ± 0.116	-1.422 ± 0.175	0.39 ± 0.05	1.00
**20**	I45	-3.161 ± 0.104			1.00
**21**	V46	-3.348 ± 0.235			1.00
**22**	**L47**	-3.418 ± 0.179			1.00
**23**	**L48**	-3.466 ± 0.261			1.00
**24**	L49	-3.607 ± 0.090	-0.551 ± 0.415	0.19 ± 0.04	1.00
**25**	V50	-3.184 ± 0.131	-1.711 ± 0.551	0.29 ± 0.09	1.00
**26**	M51	-2.640 ± 0.126	-0.609 ± 0.995	0.19 ± 0.14	1.00
**27**	L52	-2.050 ± 0.186			1.00
**28**	K53	n.d.			
**29**	K54	n.d.			
**30**	K28	n.d.			

		pL-A9 [1]						pL-GG [1]			
**Residue number**	**amide**	**log $k_{(B)}$** [2] [min^{-1}]	**log k_A** [3] [min^{-1}]	**fraction A** [4]	**D(0)** [5]	**Residue numnber**	**amide**	**log $k_{(B)}$** [2] [min^{-1}]	**log k_A** [3] [min^{-1}]	**fraction A** [4]	**D(0)** [5]
**3**	K	n.d. [6]				**3**	K	n.d. [6]			
**4**	L	n.d.				**4**	L	n.d.			
**5**	L	n.d.				**5**	L	n.d.			
**6**	L	-1.238 ± 0.147	0.084 ± 0.168	0.52 ± 0.08	1.00	**6**	L	-1.406 ± 0.115	-0.290 ± 0.258	0.38 ± 0.12	1.00
**7**	L	-1.753 ± 0.058	-0.505 ± 0.578	0.15 ± 0.09	1.00	**7**	L	-2.093 ± 0.041	-0.024 ± 0.367	0.19 ± 0.03	1.00
**8**	L	-2.867 ± 0.045	-1.750 ± 0.058	0.39 ± 0.03	1.00	**8**	L	-3.190 ± 0.075	-2.113 ± 0.145	0.32 ± 0.07	1.00
**9**	L	-3.258 ± 0.066			1.00	**9**	L	-3.362 ± 0.028			1.00
**10**	L	-3.877 ± 0.178			1.00	**10**	L	-3.403 ± 0.031			1.00
**11**	L	-4.215 ± 0.139			1.00	**11**	L	-3.247 ± 0.028	-0.768 ± 0.210	0.16 ± 0.02	1.00
**12**	L	-4.460 ± 0.061			1.00	**12**	**G**	-2.515 ± 0.063	-0.408 ± 0.145	0.26 ± 0.03	1.00
**13**	L	-4.706 ± 0.100			1.00	**13**	**G**	-2.541 ± 0.114	-1.009 ± 0.102	0.58 ± 0.05	1.00
**14**	L	-4.487 ± 0.241			1.00	**14**	L	-2.645 ± 0.350	-1.850 ± 0.150	0.58 ± 0.22	1.00
**15**	L	-4.681 ± 0.191			1.00	**15**	L	-2.986 ± 0.080			1.00
**16**	L	-4.594 ± 0.128			1.00	**16**	L	-3.243 ± 0.056			1.00
**17**	L	-4.614 ± 0.093			1.00	**17**	L	-3.324 ± 0.109			1.00
**18**						**18**	L	-3.786 ± 0.069			0.80
**19**						**19**	L	-4.198 ± 0.198			0.80
**20**						**20**	L	-4.175 ± 0.252			0.90
**21**						**21**	L	-4.433 ± 0.160			0.90
**22**						**22**	L	-4.523 ± 0.155			0.85
**23**						**23**	L	-4.478 ± 0.264			0.75
**24**						**24**	L	-3.964 ± 0.154			0.75
**25**						**25**	L	-3.528 ± 0.077			0.90
**26**						**26**	L	-3.192 ± 0.093			1.00
**27**						**27**	L	-3.245 ± 0.060	-1.054 ± 0.132	0.31 ± 0.03	1.00
**28**						**28**	K	n.d.			
**29**						**29**	K	n.d.			
**30**						**30**	K	n.d.			

		pL-VGGV [1]						pL-VGGV/εGG [1]			
**Residue number**	**amide**	**log $k_{(B)}$** [2] [min^{-1}]	**log k_A** [3] [min^{-1}]	**fraction A** [4]	**D(0)** [5]	**Residue numnber**	**amide**	**log $k_{(B)}$** [2] [min^{-1}]	**log k_A** [3] [min^{-1}]	**fraction A** [4]	**D(0)** [5]
**3**	K	n.d.[6]				**3**	K	n.d.[6]			
**4**	L	n.d.				**4**	L	n.d.			
**5**	L	n.d.				**5**	L	n.d.			
**6**	L	-1.306 ± 0.097	0.153 ± 0.575	0.24 ± 0.12	1.00	**6**	L	-1.050 ± 0.103	0.265 ± 0.300	0.30 ± 0.08	1.00
**7**	L	-2.119 ± 0.053	-1.159 ± 0.183	0.35 ± 0.09	1.00	**7**	L	-1.881 ± 0.050	-0.617 ± 0.215	0.23 ± 0.06	1.00
**8**	L	-2.728 ± 0.032			0.95	**8**	L	-2.528 ± 0.061			1.00
**9**	L	-3.146 ± 0.051			1.00	**9**	L	-3.074 ± 0.037			1.00
**10**	L	-3.157 ± 0.034			1.00	**10**	L	-3.105 ± 0.040			1.00
**11**	**V**	-3.268 ± 0.055	-1.676 ± 0.140	0.33 ± 0.04	1.00	**11**	**V**	-3.105 ± 0.052	-1.711 ± 0.151	0.29 ± 0.04	1.00
**12**	**G**	-2.229 ± 0.091	-0.258 ± 0.302	0.25 ± 0.05	1.00	**12**	**G**	-2.299 ± 0.104	-0.826 ± 0.166	0.26 ± 0.06	1.00
**13**	**G**	-1.932 ± 0.099	-0.125 ± 0.164	0.32 ± 0.05	1.00	**13**	**G**	-2.078 ± 0.064	-0.887 ± 0.101	0.34 ± 0.05	1.00
**14**	**V**	-2.526 ± 0.149	-1.175 ± 0.162	0.55 ± 0.09	1.00	**14**	**V**	-2.727 ± 0.123	-1.494 ± 0.070	0.64 ± 0.06	1.00
**15**	L	-2.610 ± 0.052			1.00	**15**	L	-2.609 ± 0.126			1.00
**16**	L	-2.889 ± 0.078			1.00	**16**	L	-2.883 ± 0.085			1.00
**17**	L	-3.243 ± 0.120			1.00	**17**	L	-3.115 ± 0.150			0.85
**18**	L	-3.619 ± 0.094			0.95	**18**	L	-3.699 ± 0.238			0.85
**19**	L	-3.839 ± 0.117			0.95	**19**	L	-4.024 ± 0.066			0.85
**20**	L	-4.134 ± 0.225			0.90	**20**	L	-3.883 ± 0.134			0.90
**21**	L	-4.372 ± 0.138			0.85	**21**	L	-3.709 ± 0.067	-1.309 ± 0.433	0.19 ± 0.03	1.00
**22**	L	-4.455 ± 0.208			0.85	**22**	L	-3.326 ± 0.124	-0.829 ± 0.283	0.34 ± 0.04	1.00
**23**	L	-4.633 ± 0.123	-3.431 ± 0.293	0.32 ± 0.11	0.85	**23**	**G**	-2.820 ± 0.111	-0.777 ± 0.091	0.56 ± 0.03	1.00
**24**	L	-4.476 ± 0.210	-3.241 ± 0.487	0.34 ± 0.18	0.90	**24**	**G**	-1.534 ± 0.171	-0.314 ± 0.187	0.45 ± 0.11	1.00
**25**	L	-4.122 ± 0.257	-2.976 ± 0.260	0.48 ± 0.17	1.00	**25**	L	-1.505 ± 0.055	0.047 ± 0.061	0.50 ± 0.03	1.00
**26**	L	-3.208 ± 0.088	-1.376 ± 0.222	0.21 ± 0.05	1.00	**26**	L	-0.848 ± 0.093			1.00
**27**	L	-3.172 ± 0.067	-0.725 ± 0.131	0.36 ± 0.03	1.00	**27**	L	-1.210 ± 0.069	0.285 ± 0.268	0.28 ± 0.05	1.00
**28**	K	n.d.				**28**	K	n.d.			
**29**	K	n.d.				**29**	K	n.d.			
**30**	K	n.d.				**30**	K	n.d.			

Residue number	amide	pL-VGGV/cr [1]			
		log $k_{(B)}$ [2] [min^{-1}]	log k_A [3] [min^{-1}]	fraction A [4]	D(0) [5]
**3**	K	n.d.[6]			
**4**	L	n.d.			
**5**	L	n.d.			
**6**	L	-1.124 ± 0.068	0.342 ± 0.214	0.34 ± 0.05	1.00
**7**	L	-1.968 ± 0.090	-0.890 ± 0.299	0.29 ± 0.10	1.00
**8**	L	-2.926 ± 0.074	-1.916 ± 0.114	0.35 ± 0.07	1.00
**9**	L	-3.130 ± 0.032			1.00
**10**	L	-3.100 ± 0.024			1.00
**11**	**V**	-3.026 ± 0.068	-0.439 ± 0.246	0.28 ± 0.03	1.00
**12**	**G**	-2.176 ± 0.176	-0.435 ± 0.379	0.31 ± 0.10	1.00
**13**	**G**	-1.759 ± 0.059	-0.162 ± 0.260	0.22 ± 0.05	1.00
**14**	**V**	-1.990 ± 0.067			1.00
**15**	L	-2.494 ± 0.070			1.00
**16**	L	-2.628 ± 0.067			1.00
**17**	L	-2.794 ± 0.067			0.85
**18**	L	-3.082 ± 0.094			0.85
**19**	**V**	-3.819 ± 0.449			0.90
**20**	**I**	-4.144 ± 0.094			1.00
**21**	**V**	-4.460 ± 0.102			1.00
**22**	**I**	-4.788 ± 0.138	-3.151 ± 0.199	0.27 ± 0.08	1.00
**23**	**T**	-4.345 ± 0.081	-1.547 ± 0.280	0.18 ± 0.03	1.00
**24**	**L**	-4.000 ± 0.085	-0.763 ± 0.145	0.23 ± 0.02	1.00
**25**	**V**	-3.366 ± 0.105	-0.560 ± 0.748	0.19 ± 0.07	1.00
**26**	**M**	-2.836 ± 0.112			1.00
**27**	**L**	-2.622 ± 0.093	-0.311 ± 0.432	0.26 ± 0.05	1.00
**28**	K	n.d.			
**29**	K	n.d.			
**30**	K	n.d.			

[1] Identity of the TMD peptide

[2] k_{exp} from mono-exponential fits or $k_{exp,B}$ of slowly exchanging population in case of biexponential fits

[3] k_{exp} of quickly exchanging population from biexponential fits

[4] fraction of deuterons that exchange with $k_{exp,A}$ in biexponential fits

[5] set number of deuterons at time = 0 min

[6] n.d. = not determined due to missing data

Supplementary Table 3. Mass and sequence of AICD peptides generated in the cell-free assay as detected by MALDI-TOF MS (part (f) in Figs. 2 - 4). Da, Dalton.

Construct	Sequence	Peptide	Calculated mass [Da]	Observed mass [Da]
**C99**				
	LVMLKKKQYTSIHHGVVEVDAAVTPEERHLSKMQQNGYENPTYKFFEQMQNGSRSHHHHHH	ε48	7235.10	7236.44
	VMLKKKQYTSIHHGVVEVDAAVTPEERHLSKMQQNGYENPTYKFFEQMQNGSRSHHHHHH	ε49	7121.94	7124.44
	LKKKQYTSIHHGVVEVDAAVTPEERHLSKMQQNGYENPTYKFFEQMQNGSRSHHHHHH	ε51	6891.61	6887.77
**pL-GG**				
	LLLLKKKQYTSIHHGVVEVDAAVTPEERHLSKMQQNGYENPTYKFFEQMQNGSRSHHHHHH	ε48	7231.09	7232.41
	LLLKKKQYTSIHHGVVEVDAAVTPEERHLSKMQQNGYENPTYKFFEQMQNGSRSHHHHHH	ε49	7117.93	7116.61
**pL-VGGV**				
	LLLLKKKQYTSIHHGVVEVDAAVTPEERHLSKMQQNGYENPTYKFFEQMQNGSRSHHHHHH	ε48	7231.09	7227.85
	LLLKKKQYTSIHHGVVEVDAAVTPEERHLSKMQQNGYENPTYKFFEQMQNGSRSHHHHHH	ε49	7117.93	7121.17
**pL-cr**				
	LVMLKKKQYTSIHHGVVEVDAAVTPEERHLSKMQQNGYENPTYKFFEQMQNGSRSHHHHHH	ε48	7235.10	7234.45
	VMLKKKQYTSIHHGVVEVDAAVTPEERHLSKMQQNGYENPTYKFFEQMQNGSRSHHHHHH	ε49	7121.94	7122.59
**pL-VGGV/cr**				
	LVMLKKKQYTSIHHGVVEVDAAVTPEERHLSKMQQNGYENPTYKFFEQMQNGSRSHHHHHH	ε48	7235.10	7236.18
	VMLKKKQYTSIHHGVVEVDAAVTPEERHLSKMQQNGYENPTYKFFEQMQNGSRSHHHHHH	ε49	7121.94	7120.86
**pL-VGGV/ εGG**				
	GLLLKKKQYTSIHHGVVEVDAAVTPEERHLSKMQQNGYENPTYKFFEQMQNGSRSHHHHHH	ε48	7174.98	7175.70

Table S4. Mass and sequence of Aβ peptides generated in the cell-free assay as detected by MALDI-TOF MS (part (g) in Figs. 2 - 4). Da, Dalton.

Construct	Sequence	Peptide	Calculated mass [Da]	Observed mass [Da]
**C99**				
	MDAEFRHDSGYEVHHQKLVFFAEDVGSNK GAIIGLMVG	Aβ37	4207.73	4207.75
	MDAEFRHDSGYEVHHQKLVFFAEDVGSNK GAIIGLMVGG	Aβ38	4264.79	4263.75
	MDAEFRHDSGYEVHHQKLVFFAEDVGSNK GAIIGLMVGGV	Aβ39	4363.92	4366.16
	MDAEFRHDSGYEVHHQKLVFFAEDVGSNK GAIIGLMVGGVV	Aβ40	4463.05	4462.96
	MDAEFRHDSGYEVHHQKLVFFAEDVGSNK GAIIGLMVGGVVIA	Aβ42	4647.29	4646.17
**pL-GG**				
	MDAEFRHDSGYEVHHQKLVFFAEDVGSNK LLLLLL	Aβ34	4074.65	4075.64
**pL-VGGV**				
	MDAEFRHDSGYEVHHQKLVFFAEDVGSNK LLLLLL	Aβ34	4074.65	4073.86
	MDAEFRHDSGYEVHHQKLVFFAEDVGSNK LLLLLLL	Aβ35	4187.81	4188.24
	MDAEFRHDSGYEVHHQKLVFFAEDVGSNK LLLLLLLVG	Aβ37	4343.99	4345.07
	MDAEFRHDSGYEVHHQKLVFFAEDVGSNK LLLLLLLVGG	Aβ38	4401.05	4400.33
**pL-cr**				
	MDAEFRHDSGYEVHHQKLVFFAEDVGSNK LLLLLLLL	Aβ36	4300.97	4301.11
	MDAEFRHDSGYEVHHQKLVFFAEDVGSNK LLLLLLLLL	Aβ37	4414.13	4414.79
	MDAEFRHDSGYEVHHQKLVFFAEDVGSNK LLLLLLLLLL	Aβ38	4527.29	4526.49
**pL-VGGV/cr**				
	MDAEFRHDSGYEVHHQKLVFFAEDVGSNK LLLLLL	Aβ34	4074.65	4073.94
	MDAEFRHDSGYEVHHQKLVFFAEDVGSNK LLLLLLL	Aβ35	4187.81	4187.43
	MDAEFRHDSGYEVHHQKLVFFAEDVGSNK LLLLLLLVG	Aβ37	4343.99	4344.49
	MDAEFRHDSGYEVHHQKLVFFAEDVGSNK LLLLLLLVGG	Aβ38	4401.05	4401.04
	MDAEFRHDSGYEVHHQKLVFFAEDVGSNK LLLLLLLVGGV	Aβ39	4500.18	4500.78

Construct	Sequence	Peptide	Calculated mass [Da]	Observed mass [Da]
**pL-VGGV/ εGG**				
	MDAEFRHDSGYEVHHQKLVFFAEDVGSNK LLLLLL	Aβ34	4074.65	4072.93
	MDAEFRHDSGYEVHHQKLVFFAEDVGSNK LLLLLLL	Aβ35	4187.81	4188.19
	MDAEFRHDSGYEVHHQKLVFFAEDVGSNK LLLLLLLVG	Aβ37	4343.99	4343.20
	MDAEFRHDSGYEVHHQKLVFFAEDVGSNK LLLLLLLVGG	Aβ38	4401.05	4403.19

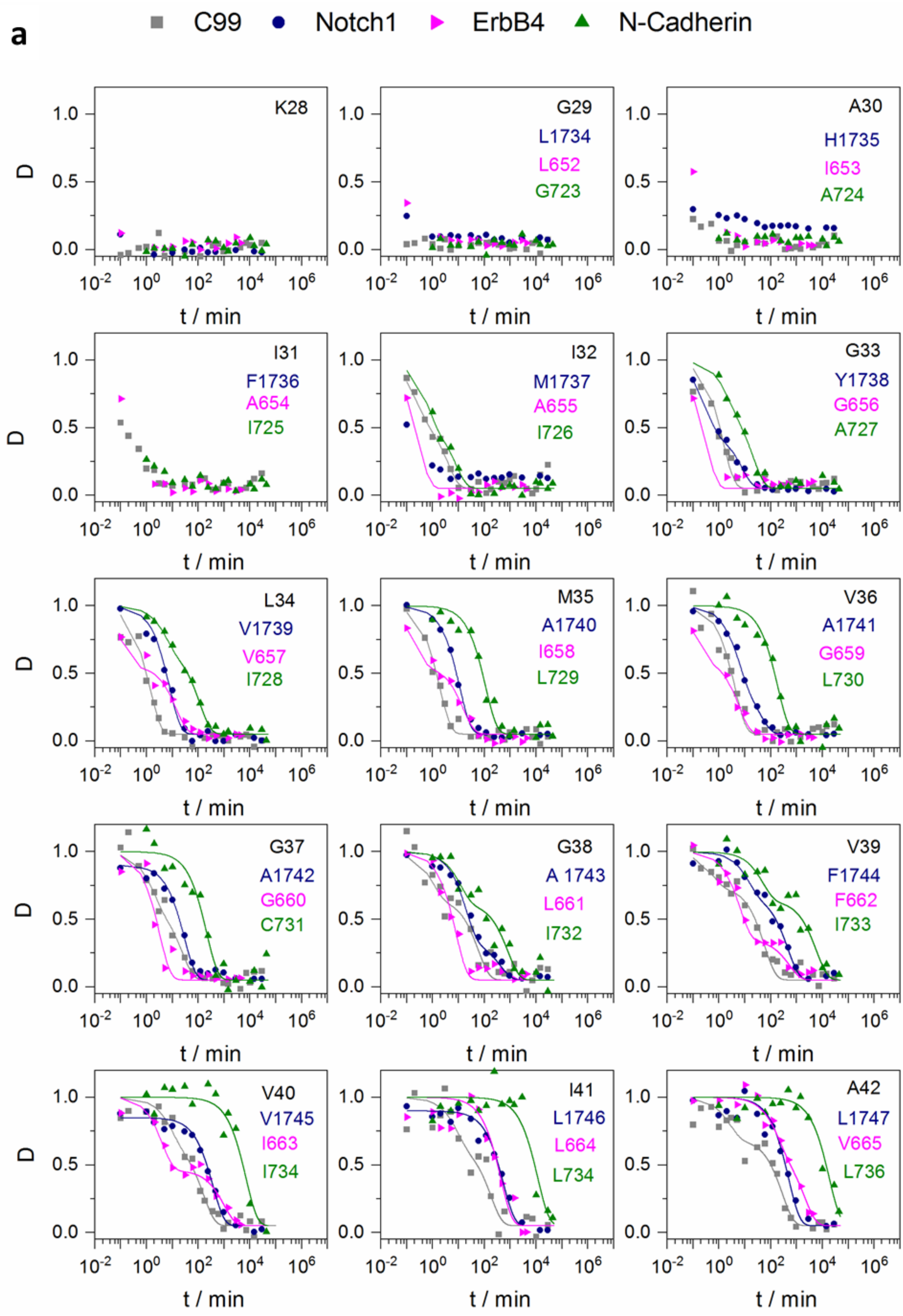
a
C99
Notch1
ErbB4
N-Cadherin
K28
G29
L1734
L652
G723
A30
H1735
I653
A724
I31
F1736
A654
I725
I32
M1737
A655
I726
G33
Y1738
G656
A727
L34
V1739
V657
I728
M35
A1740
I658
L729
V36
A1741
G659
L730
G37
A1742
G660
C731
G38
A 1743
L661
I732
V39
F1744
F662
I733
V40
V1745
I663
I734
I41
L1746
L664
L734
A42
L1747
V665
L736
D
t / min

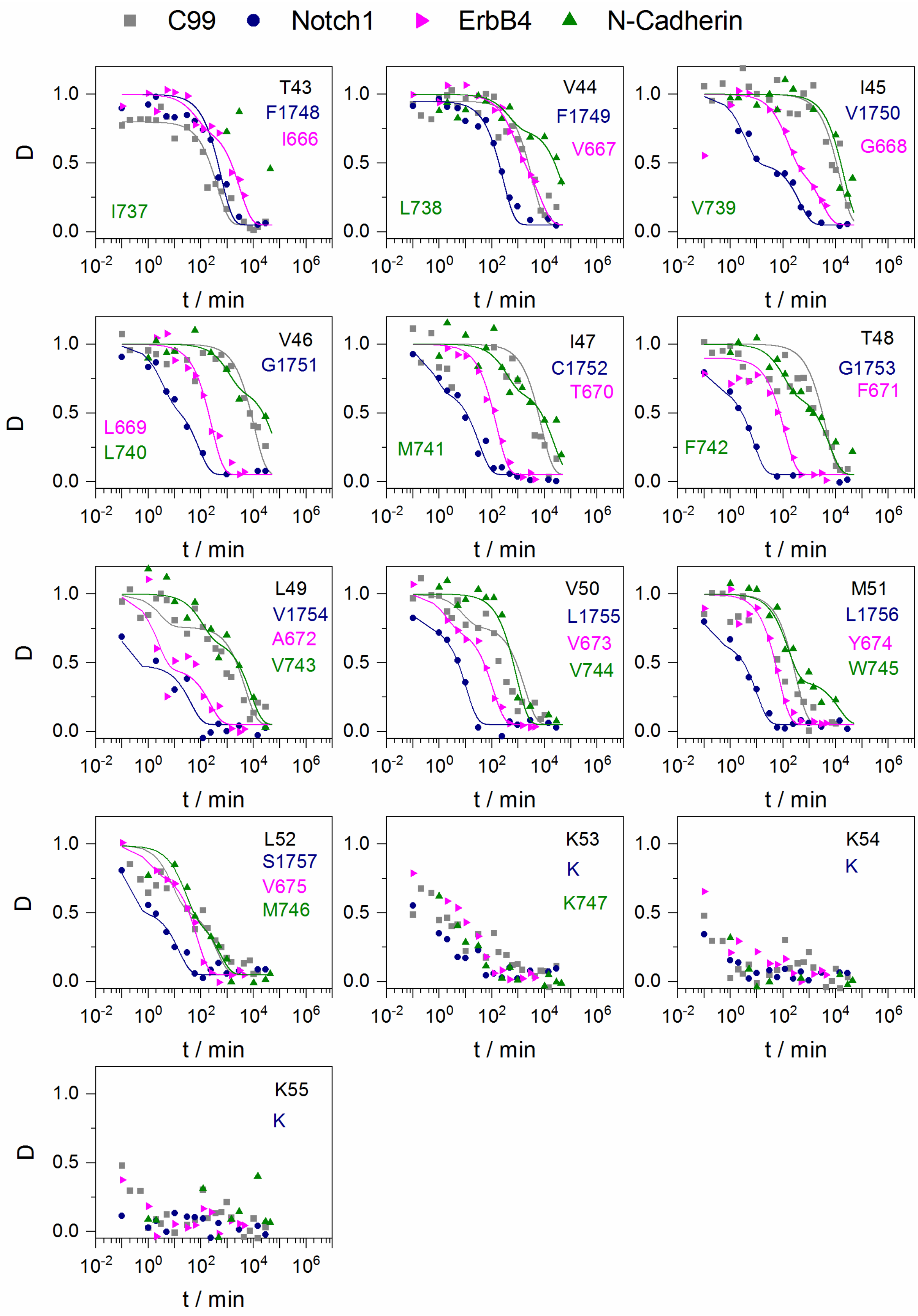
C99
Notch1
ErbB4
N-Cadherin
T43
F1748
I666
I737
V44
F1749
V667
L738
I45
V1750
G668
V739
V46
G1751
L669
L740
I47
C1752
T670
M741
T48
G1753
F671
F742
L49
V1754
A672
V743
V50
L1755
V673
V744
M51
L1756
Y674
W745
L52
S1757
V675
M746
K53
K
K747
K54
K
K55
K
D
t / min

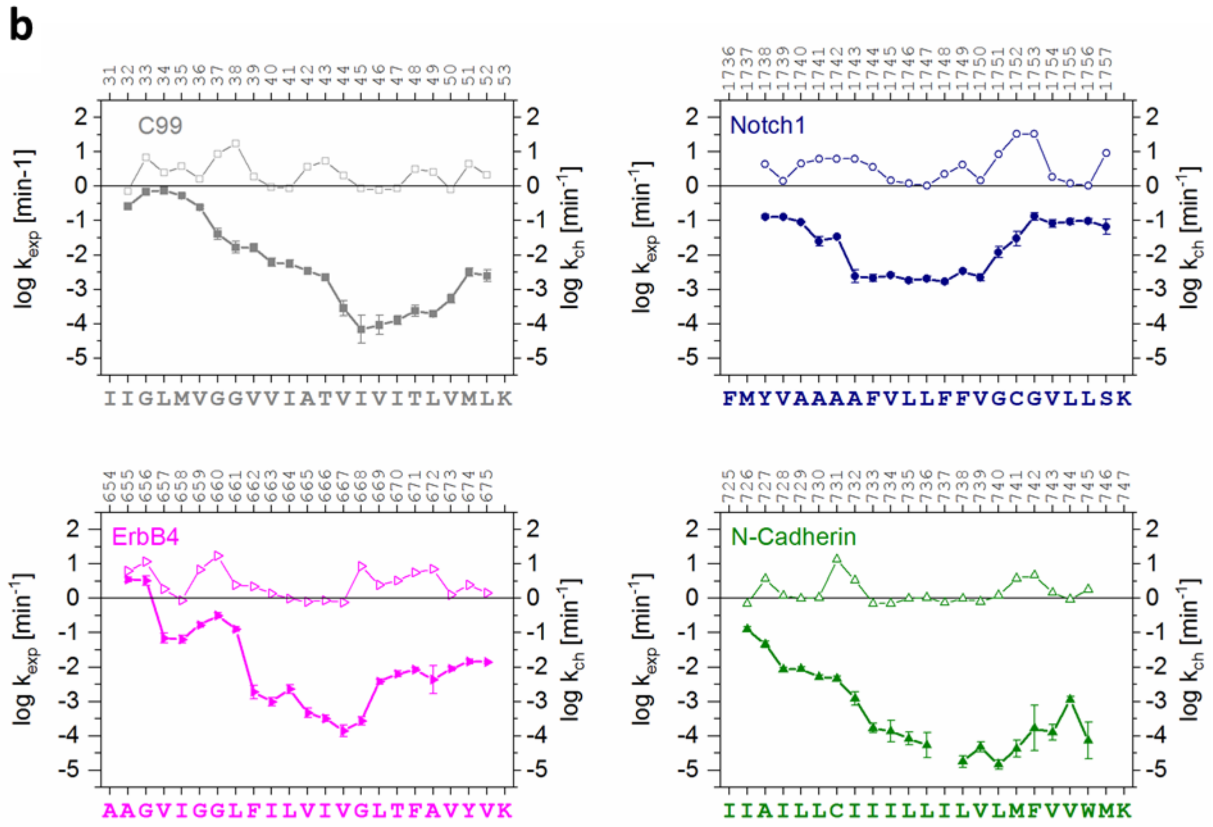

Supplementary Figure 1. DHX of C99, Notch1, ErbB4, and N-Cadherin TMDs. (a) Residue-specific amide DHX kinetics where the calculated deuterium contents D of the respective amides are plotted against the exchange period t. The kinetics are overlayed after aligning the sequences at the terminal Lys-tags. **(b)** Exchange rate constants k_{exp} of individual amide deuterons (filled symbols, N = 3, $\log k_{exp}$ ± error of fit) and chemical exchange rate constants k_{ch} (emtpy symbols). Values of C99 and Notch1 were obtained after reevaluating data from refs. [8,10,11] after supplementing them with additional measurements to increase the density of the data points.

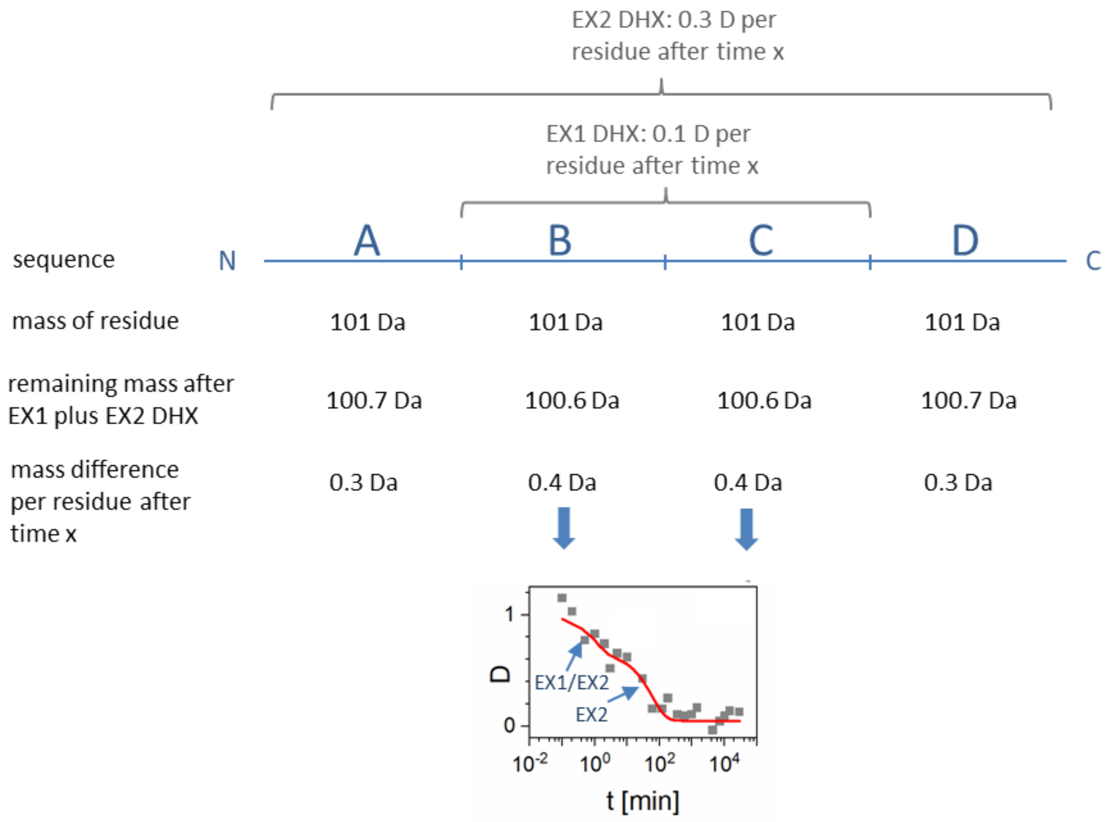

Supplementary Figure 2. A model case illustrating how a local contribution of EX1 DHX to amide exchange can artificially accelerate exchange at early time points of the reaction. For simplicity, we assume a peptide of four residues (A – D) where each residue has a mass of 101 Da after deuteration of its amide. After a given period x of exchange we assume that residues A and D exchange 0.3 D by EX2 while residues B and C exchange a total of 0.4 D by low abundance EX1 (0.1 D) superimposed onto prevalent EX2 (0.3 D). The mass differences at the different residues are obtained by subtraction of the different c and z fragments (as obtained by ETD) from each others. Note the higher mass difference of 0.4 D at residues B and C which is expected to lead to an accelerated exchange rate constant k_{exp} calculated for an early phase of DHX, thus generating a biphasic shape of the kinetics (exemplified by that at C99 G38 taken from Fig. S3). At later time points, DD neighbors will have been replaced by DH neighbors where EX1 DHX cannot be distinguished from EX2.

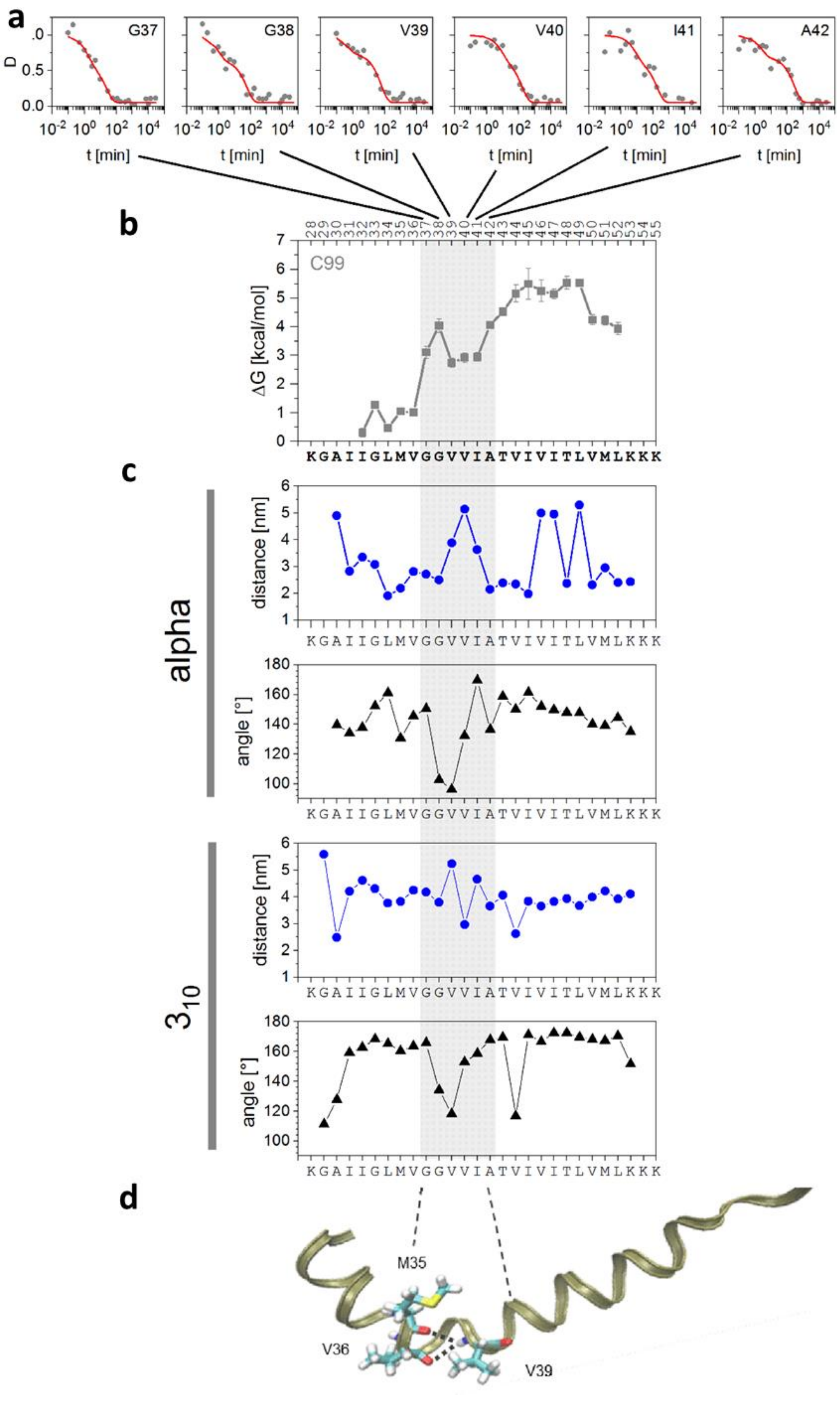
a
G37
G38
V39
V40
I41
A42
D
t [min]
b
C99
ΔG [kcal/mol]
KGAIIGLMVGGVVIATVIVITLVMLKKK
c
alpha
distance [nm]
angle [°]
3
10
d
M35
V36
V39

Supplementary Figure 3. Biexponential DHX and amide H-bonding of wt C99. (a) Exemplary biphasic DHX kinetics near the hinge region (reproduced from Fig. S1). **(b)** H-bond stabilities ΔG (reproduced from Fig. 1). **(c)** Potential α (i,i-4) and 3_{10} (i,i-3) helical amide H-bond geometries calculated from the first model of NMR structure pdb 2lp1 [12]. Yellow shading marks residues giving rise to biphasic DHX, as illustrated in part (a). **(d)** First model of pdb 2lp1 from K28 to K55. Note that the N-H ... O=C distances (broken lines) and angles between V39 and M35 (d=3.88 Å, Θ= 96°) or V36 (d=5.23 Å, Θ= 118°), respectively, indicate extremely weak potential amide H-bonds originating from V39 that exhibits the most pronounced biphasic DHX (model generated with VMD [13]).

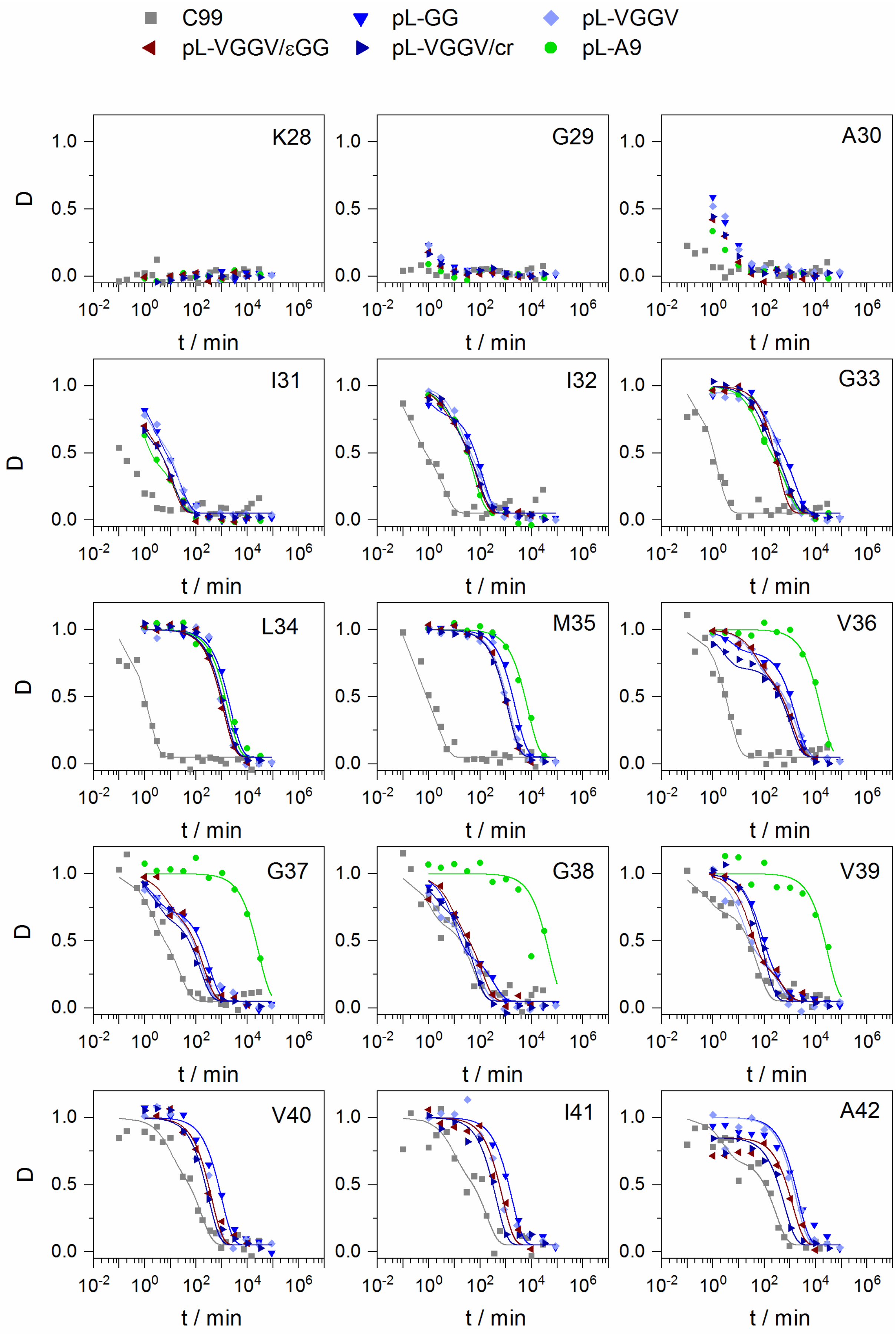
C99
pL-GG
pL-VGGV
pL-VGGV/εGG
pL-VGGV/cr
pL-A9
K28
G29
A30
I31
I32
G33
L34
M35
V36
G37
G38
V39
V40
I41
A42
D
t / min

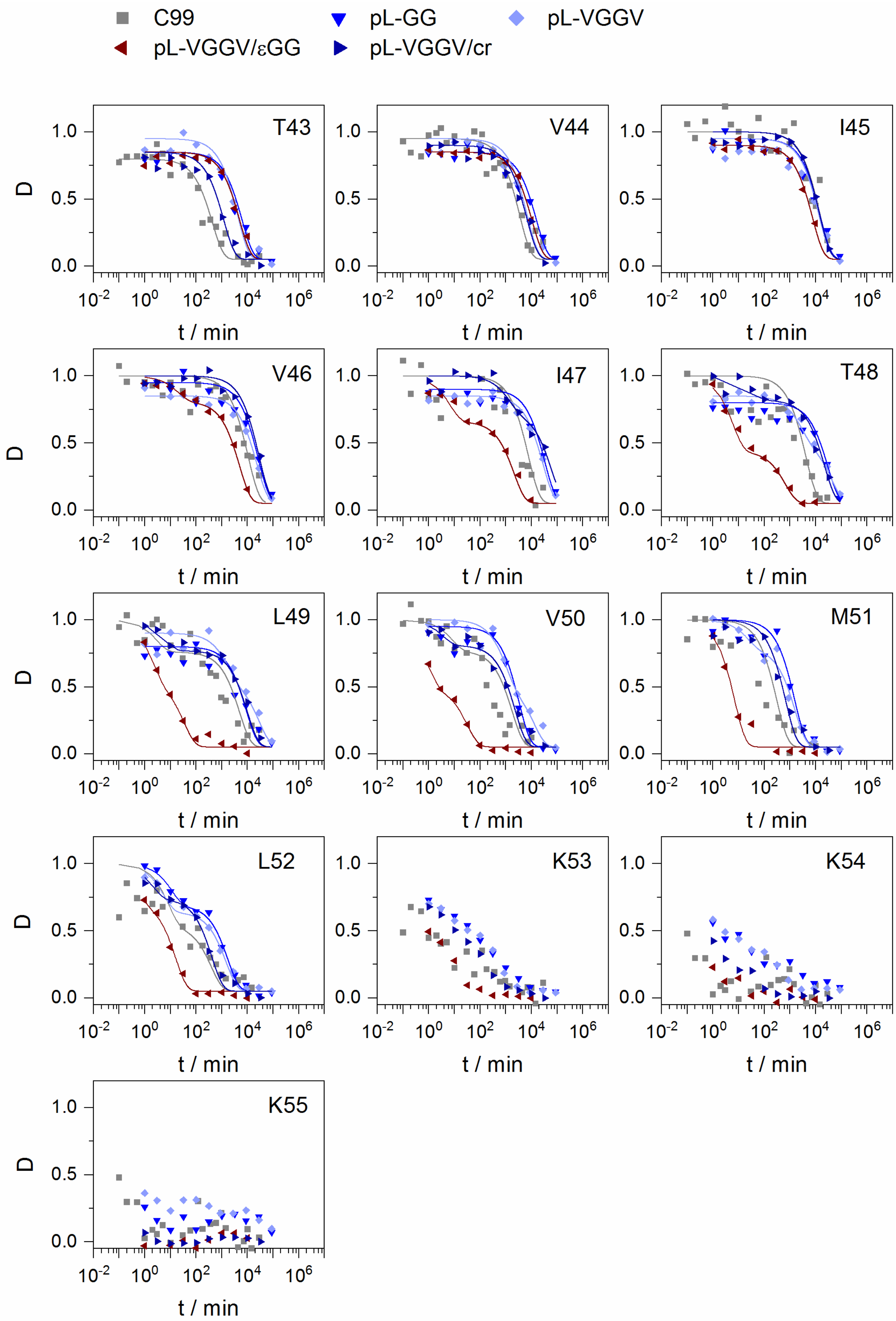
C99
pL-GG
pL-VGGV
pL-VGGV/εGG
pL-VGGV/cr
T43
V44
I45
V46
I47
T48
L49
V50
M51
L52
K53
K54
K55
D
t / min

Supplementary Figure 4. Residue-specific amide DHX kinetics of C99 derivatives. The calculated deuterium contents D (mean values, N = 3) of the respective amides are plotted against the exchange period t. The shown kinetics were used to calculate the respective k_{exp} values after data fitting with monoexponential or biexponential decay functions. Fitting was only performed for those kinetics that were deemed complete enough for calculating k_{exp}. Sequence positions (Aβ numbering) are given in the insets; the amino acid type at a given position corresponds to the wt C99 sequence. C99 data are taken from Fig. S1.

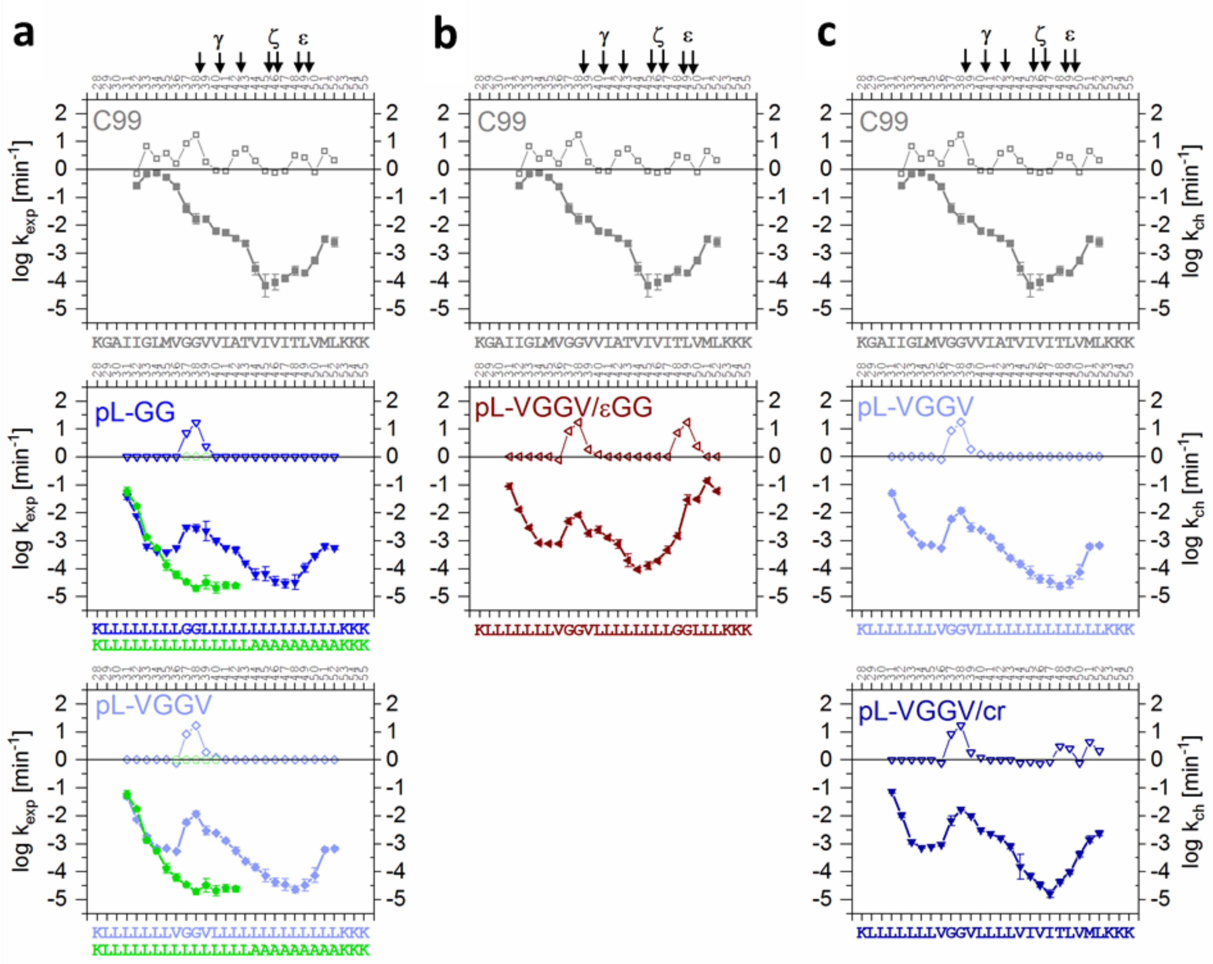

Supplementary Figure 5. DHX rate constants of TMD peptides. Rate constants k_{exp} of constructs whose ΔG values are depicted in Fig. 2 **(a)**, Fig. 3 **(b)**, or Fig. 4 **(c)**. Shown are k_{exp} of individual amide deuterons (filled symbols, N = 3 independent DHX reactions, log k_{exp} ± error of fit) and the respective amide-specific intrinsic chemical exchange rate constants k_{ch} that describe the exchange kinetics in the unfolded state [14] (emtpy symbols). Note that diverging values of k_{exp} and k_{ch} indicate folded regions that are partially protected from exchange.

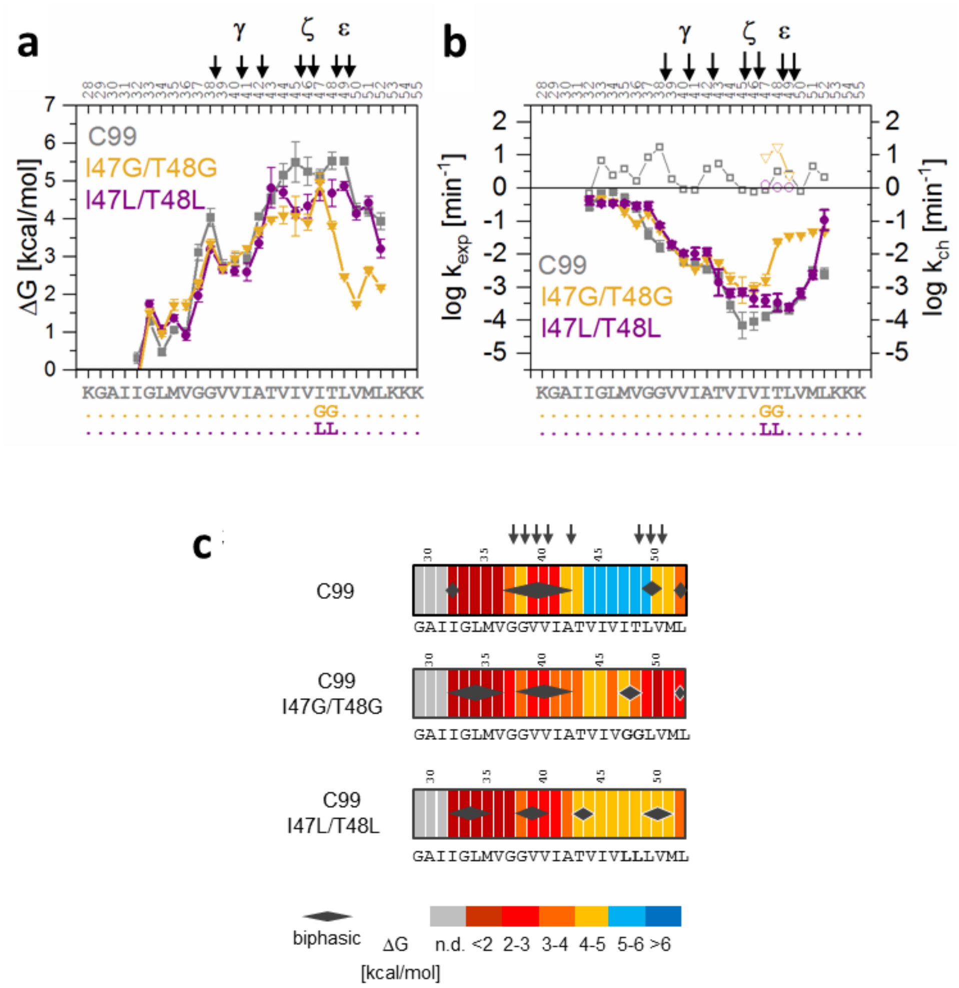

a
b
c
γ
ζ
ε
C99
I47G/T48G
I47L/T48L
ΔG [kcal/mol]
log k$_{exp}$ [min^{-1}]
log k$_{ch}$ [min^{-1}]
KGAIIGLMVGGVVIATVIVITLVMLKKK
GG
LL
C99
GAIIGLMVGGVVIATVIVITLVML
C99 I47G/T48G
GAIIGLMVGGVVIATVIVGGLVML
C99 I47L/T48L
GAIIGLMVGGVVIATVIVLLLVML
biphasic
ΔG [kcal/mol]
n.d. <2 2-3 3-4 4-5 5-6 >6

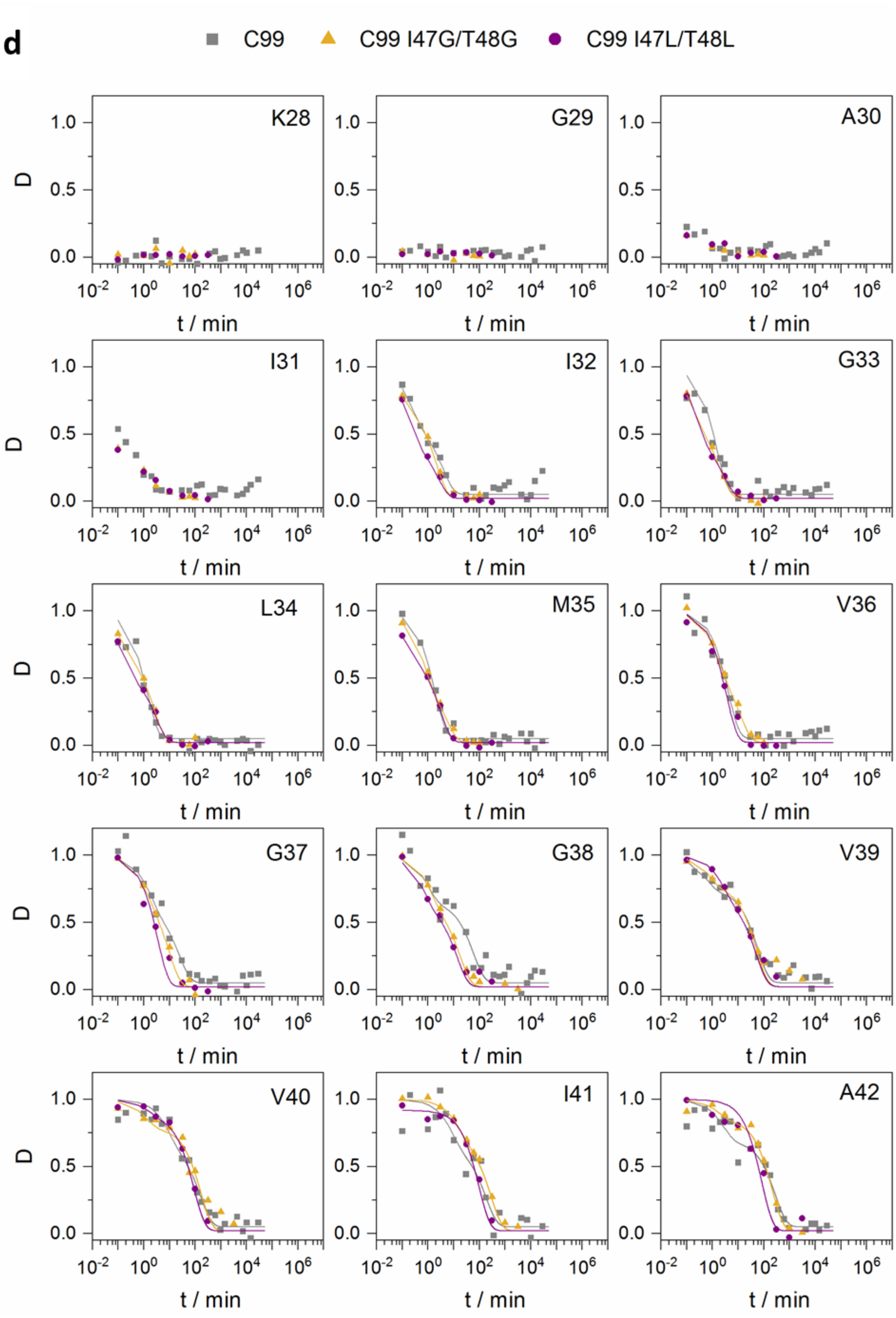
d
C99
C99 I47G/T48G
C99 I47L/T48L
K28
G29
A30
I31
I32
G33
L34
M35
V36
G37
G38
V39
V40
I41
A42
D
t / min

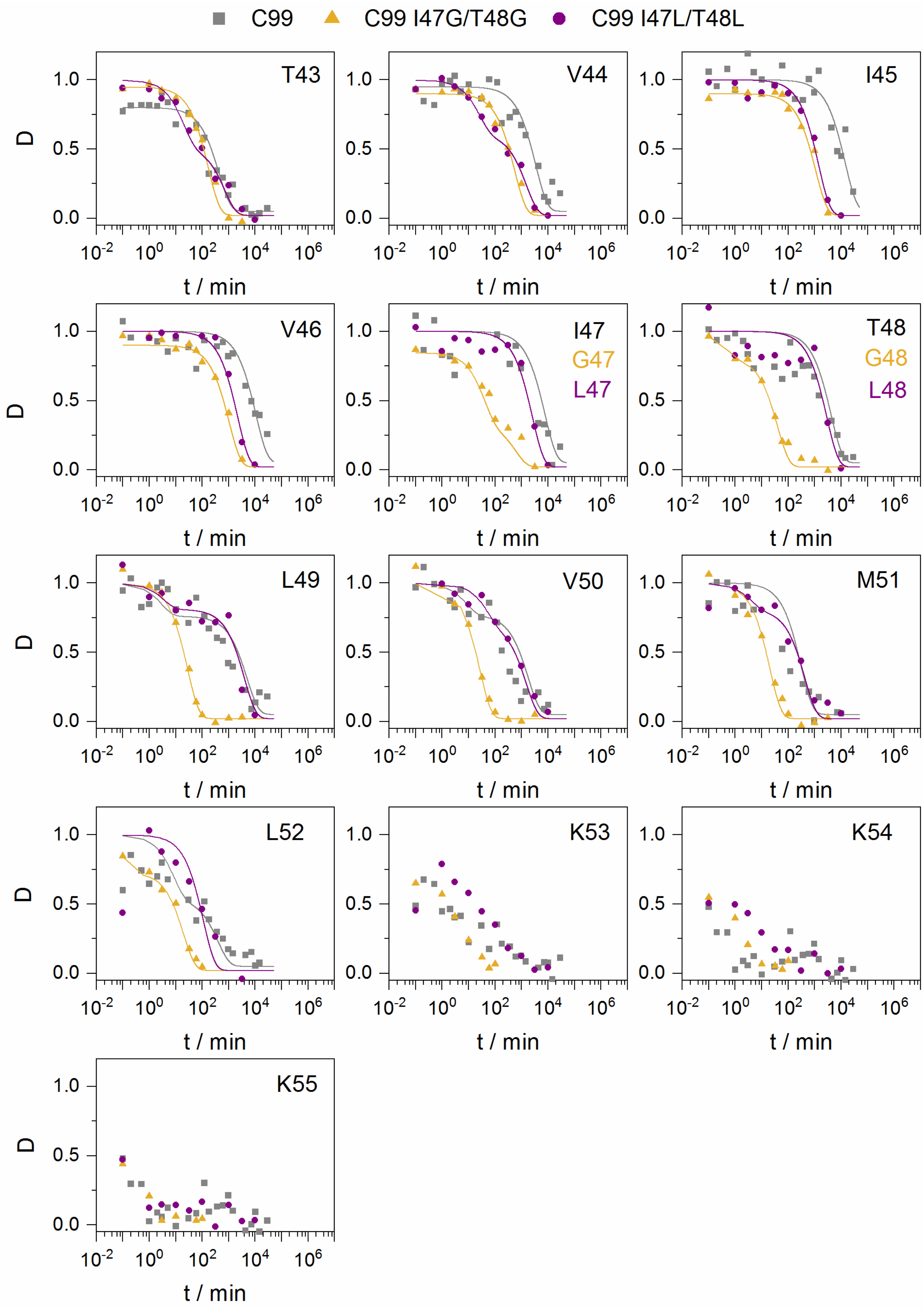

C99
C99 I47G/T48G
C99 I47L/T48L
T43
V44
I45
V46
I47
G47
L47
T48
G48
L48
L49
V50
M51
L52
K53
K54
K55
D
t / min

Supplementary Figure 6. Conformational flexibility of C99 I47G/T48G and I47L/T48L mutants. (a) Amide H-bond stabilities ΔG. The sequences feature two additional Lys residues at the N-terminus not shown here (Table S1). Aβ numbering is used and main cleavage sites are indicated. Error bars correspond to standard confidence intervals (calculated from the errors of fit in k_{exp} determination, in some cases smaller than the symbols, N=3 independent DHX reactions). **(b)** Exchange rate constants k_{exp} (filled symbols, N = 3, log k_{exp} ± error of fit) and chemical exchange rate constants k_{ch} (empty symbols). **(c)** Heat map summarizing the color-coded ΔG values, the occurence of biphasic exchange (diamonds) and canonical γ-secretase cleavage sites (arrows). **(d)** Residue-specific amide DHX kinetics where the calculated deuterium contents D of the respective amides are plotted against the exchange period t. Wt C99 data are reproduced from Fig. S1.

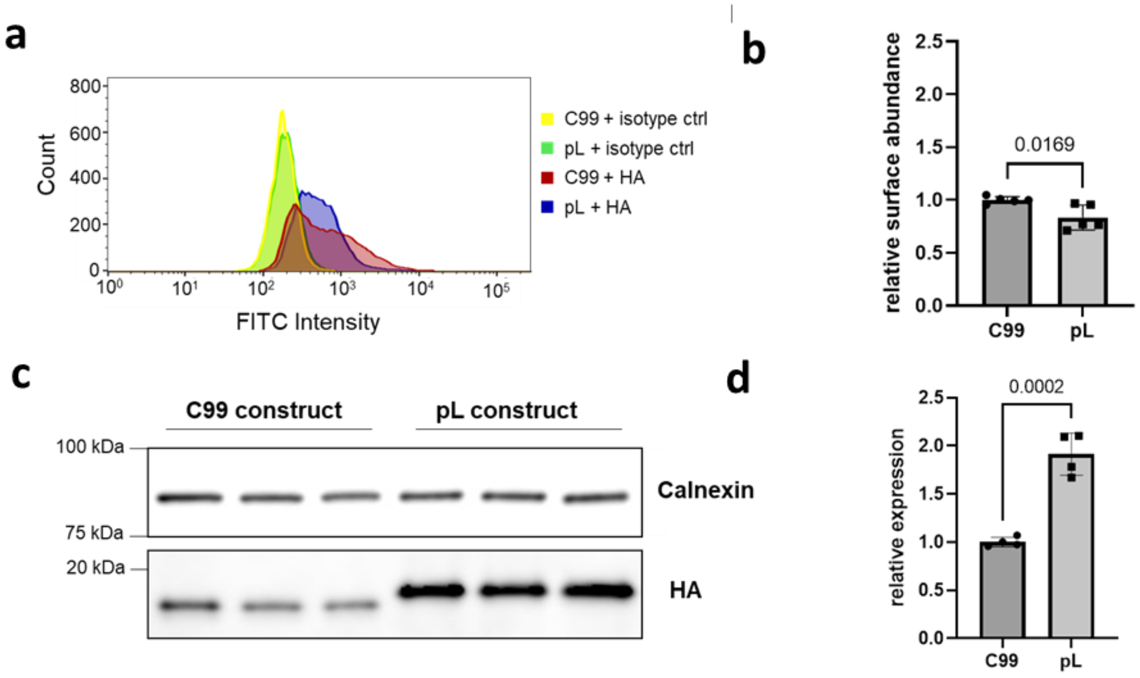

Supplementary Figure 7. Comparing cell surface localization of wt C99 and its pL derivative. (a) HEK293E cells were transfected with the respective constructs. The treated cells were suspended and labeled with HA antibody that targets the extracellular site of the constructs, or isotype control (ctrl). Shown are representative histograms from N=5 experiments. (b) The relative surface abundance of the measurement from part (a). The geometric mean of the HA-signal was divided by the respective geometric mean of the Ig-signal to obtain the mean fluorescent intensity. The C99 surface signal was used as baseline, and its average normalized to 1. A two-tailed unpaired t-test was used. (c) Cells measured in part (a) were collected, lysed and blotted against the HA-tag or calnexin as loading control. Shown are representative blots from N=4 experiments. (d) Quantification of blots in part (c). The C99 protein expression was used as baseline, and its average normalized to 1. A two-tailed unpaired t-test was used.

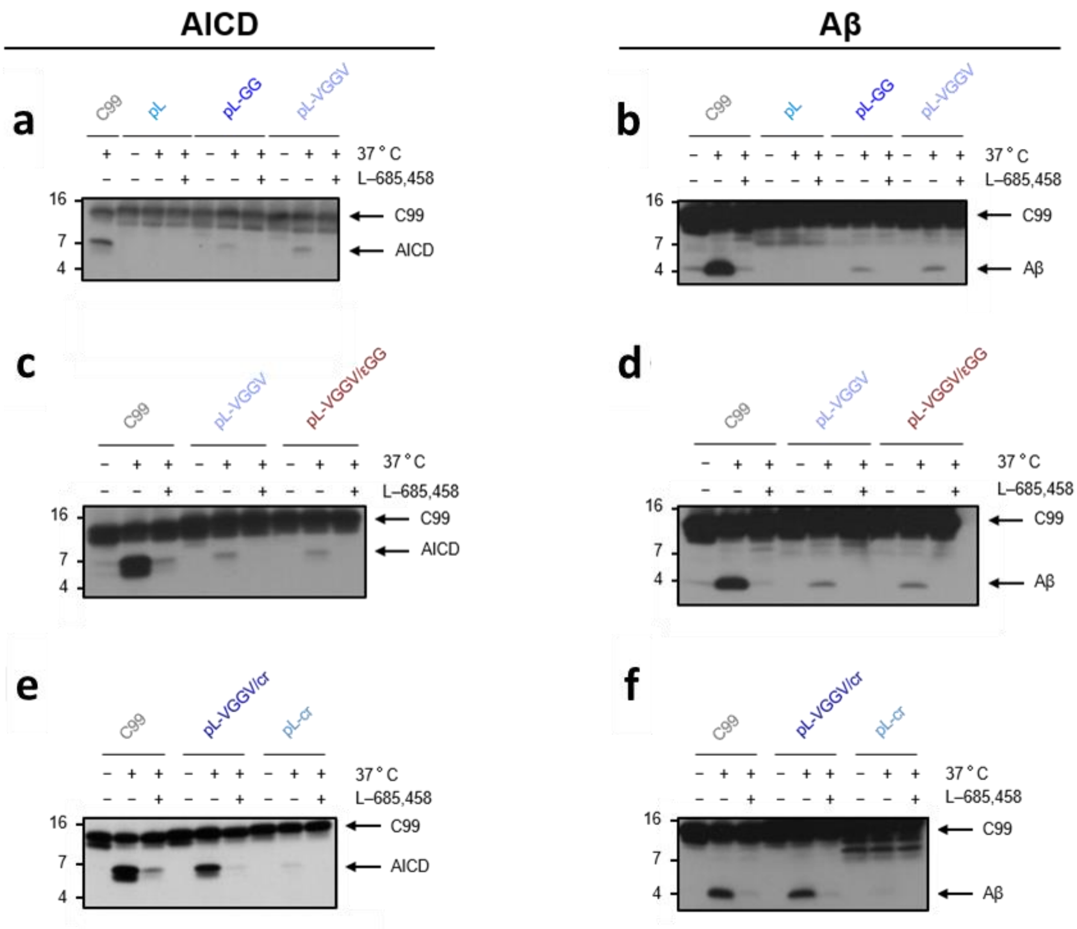

Supplementary Figure 8. Cleavage detected *in vitro* is γ-secretase dependent. (a - F) Cleavage efficiency of the different constructs after incubation with CHAPSO-solubilized HEK293 membrane fractions at 37°C. To exclude γ-secretase independent cleavage additional samples, incubated at 4°C, or at 37°C in the presence of the specific γ-secretase inhibitor L-685,458 (0.5 µM), are shown for all constructs used in this paper. Levels of AICD (a, c, e) and Aβ (b, d, f) were analyzed by immunoblotting.

Supplementary References

1 Zheng, J., Strutzenberg, T., Pascal, B. D. & Griffin, P. R. Protein dynamics and conformational changes explored by hydrogen/deuterium exchange mass spectrometry. *Curr Opin Struct Biol* **58**, 305-313, doi:10.1016/j.sbi.2019.06.007 (2019).

2 Pester, O. *et al.* The Backbone Dynamics of the Amyloid Precursor Protein Transmembrane Helix Provides a Rationale for the Sequential Cleavage Mechanism of γ-Secretase. *J. Am. Chem. Soc.* **135**, 1317-1329 (2013).

3 Ferraro, D. M., Lazo, N. D. & Robertson, A. D. EX1 Hydrogen Exchange and Protein Folding. *Biochemistry* **43**, 587-594 (2004).

4 Xiao, H. *et al.* Mapping protein engergy landscapes with amide hydrogen exchange and mass spectrometry: I. A generalized model for a two-state protein and comparison with experiment. *Prot. Sci.* **14**, 543-557 (2005).

5 Qian, H. & Chan, S. I. Hydrogen exchange kinetics of proteins in denaturants: a generalized two-process model. *J Mol Biol* **286**, 607-616, doi:S0022-2836(98)92484-6 [pii] 10.1006/jmbi.1998.2484 (1999).

6 Teilum, K., Kragelund, B. B. & Poulsen, F. M. *Application of Hydrogen Exchange Kinetics to Studies of Protein Folding*. Vol. Part I (Wiley-VCH, 2005).

7 Stelzer, W., Scharnag, C., Leurs, U., Rand, K. D. & Langosch, D. The impact of the 'Austrian' mutation of the amyloid precursor protein transmembrane helix is communicated to the hinge region. *Chemistry Select* **1**, 4408-4412 (2016).

8 Gotz, A. *et al.* Increased H-Bond Stability Relates to Altered epsilon-Cleavage Efficiency and Abeta Levels in the I45T Familial Alzheimer's Disease Mutant of APP. *Sci Rep* **9**, 5321, doi:10.1038/s41598-019-41766-1 (2019).

9 Yin, Y. I. *et al.* {gamma}-Secretase Substrate Concentration Modulates the Abeta42/Abeta40 Ratio: IMPLICATIONS FOR ALZHEIMER DISEASE. *J Biol Chem* **282**, 23639-23644, doi:10.1074/jbc.M704601200 (2007).

10 Gotz, A. *et al.* Modulating hinge flexibility in the APP transmembrane domain alters γ-secretase cleavage. *Biophys. J.* **116**, 1-18 (2019).

11 Stelzer, W. & Langosch, D. Conformationally Flexible Sites within the Transmembrane Helices of Amyloid Precursor Protein and Notch1 Receptor. *Biochemistry* **58**, 3065-3068, doi:10.1021/acs.biochem.9b00505 (2019).

12 Barrett, P. J. *et al.* The amyloid precursor protein has a flexible transmembrane domain and binds cholesterol. *Science* **336**, 1168-1171, doi:336/6085/1168 [pii] 10.1126/science.1219988 (2012).

13 Humphrey, W., Dalke, A. & Schulten, K. VMD: visual molecular dynamics. *Journal of molecular graphics* **14**, 33-38, 27-38 (1996).

14 Englander, S. W. Hydrogen exchange and mass spectrometry: A historical perspective. *J Am Soc Mass Spectrom* **17**, 1481-1489, doi:10.1016/j.jasms.2006.06.006 (2006).
